# Ten simple rules for humane data science

**DOI:** 10.1371/journal.pcbi.1011698

**Published:** 2023-12-21

**Authors:** Hassan Masum, Philip E. Bourne

**Affiliations:** 1 Waterloo Institute for Complexity and Innovation, Waterloo, Canada; 2 School of Data Science, University of Virginia, Virginia, United States of America; Carnegie Mellon University, UNITED STATES

The capabilities of data science can help us see hidden patterns, customize services, and advance biomedicine and science. As data science permeates industry and academia, a question that often arises is: How can we use these capabilities to genuinely help the world?

Considering this question brings to mind terms such as “responsible data science” and “data for good.” Inherent in these terms is the desire that data science improve the human condition—in other words, the desire to undertake humane data science.

How can we do this? What follows are 10 simple rules to contemplate.

(Note that we are referring to data science in a holistic way. Along with machine learning and AI methods, we include all aspects of the data acquisition, analysis, dissemination, and application pipeline, and their accompanying human and socioeconomic factors.)

## Rule 1: Know and follow the rules

Privacy, security, and accountability are integral to applying data science responsibly. Do you know which rules, regulations, policies, and laws apply to your work? Do you understand how to apply them?

While complying with regulations can be complex, one upside is that you can learn a lot from codified good practices. Read the text of key regulations. This can help you understand their intent and apply them effectively. Consult compliance offices and legal advisors if available (e.g., within your office of research).

Requirements from funders or institutional regulators often add rules to follow beyond what the law requires of everyone. As a case in point, major clinical and translational research funders like the NIH (the US National Institutes of Health) have data management and sharing policies that promote research reproducibility and data privacy, de-identification, quality, and reuse. Informed guidance can help navigate and implement these policies [1].

As your knowledge of rules and regulations grows, educate your colleagues on what compliance requires and why it should be prioritized. To build support for strong compliance, socialize its upside (such as security and reputational advantages).

Once you have done that…

## Rule 2: Go beyond compliance

Suppose you follow Rule 1 and meet your mandated duties and regulatory baseline. Are you willing to go beyond this?

If so, get familiar with aspirational guidelines for humane data science and AI that genuinely helps people and improves the human condition (e.g., [2]). What codes of conduct and ethical considerations are used by societies you are part of, or by organizations and people you admire? Guidelines are not all equal, so look for signals of trust and credibility, and do your own due diligence.

Consider customizing a code of conduct for your team or organization. These can be short, as long as they matter—better 1 guideline taken to heart than 10 forgotten. As noted in [3], “designing codes of conduct makes researchers more successful” since issues can be addressed thoughtfully in advance. (We encourage you to read that article for a Ten Simple Rules take on encouraging ethical practices in scientific and engineering big data research.)

Bring your guidelines alive by applying them. Discuss tough choices and engage with the consequences of your work. For example, computational biology and epidemiology can give detailed warnings of disease threats—but what of the risk of harm to people, businesses, and communities falsely identified as affected [4]?

Hold yourself accountable for your work’s social impact—avoiding harms and also seeking positive impact. Treat this impact as one way you judge your work’s success. Encourage others to do the same.

Much has been said about how to responsibly harness the powers of data science [5]. Read a representative sample and understand key themes like fairness, accountability, explainability, and safety. Connect these themes to good practices and examples in your field. For example, can you prioritize diversity in data collection to maintain equity in biomedical research and development [6]? Can that be paired with meaningful learning with and involvement by researchers from places that provide data?

This will help you to…

## Rule 3: Learn—and teach

Data science is complex and that may be even more true of its humane use. Can you survey the breadth of issues and master at least one deeply? Can you learn by doing, as you address the issues which matter most to you?

Doing good humane data science implies doing good science, such as learning good statistical practices and their nuances in your field. For example, variability of biological measurements can be amplified by many factors like a changed data collection protocol, and this needs to be accounted for [7].

Help others to understand what you learn. For example, can you clarify when a potential solution fails, what fairness or regulatory risks it might have, and what mitigation tradeoffs exist?

As you learn, teach. Build playbooks with actionable recipes to help others implement guidelines like “ensure good data governance.” At least annually, introduce guidelines to new team members, and provide refreshers and new developments for your organization and suitable partners. Consider other teaching methods like case studies, apprenticeships, active learning, simulations, failure sharing, debates, and guest speakers.

Once you have wisdom worth sharing, pass on its essence [8]. Be a bridge connecting wise practitioners with eager learners. Seek to spark or support wise leadership in others.

Find ways to apply ethics frameworks for technology and data [9]. Methods applied by prior generations may help, like the Asilomar guidelines for recombinant DNA, or approaches to the diffusion of innovations [10]. It’s also critical to keep abreast of new developments and sensitivities, such as by following trusted resources and periodically inviting external review of your practices (see Rule 5).

All of this can give you and those you teach thoughtful ways to choose wisely—and to…

## Rule 4: Act user-first

Model the users and intended beneficiaries of your work as friends to be helped, rather than pawns to be exploited. What do your users and beneficiaries need to thrive? How well do you provide that? Where do you fall short, and what biases do you bring?

Zook and colleagues suggest that we “acknowledge that data are people and can do harm”—and give examples where data that seems benign can reveal sensitive information, such as inferring the heart rates of people from analyzing videos in which they appear [3]. Remember the real people behind your data points.

Think through what acting user-first means in particular situations. For example, what should you do if someone (or some group) no longer wants their personal data used? How do you handle removing the raw data, versus handling derived data like a learned model? What if that learned model is already being relied upon by other people? Dilemmas like these can highlight issues of trust, consent, balancing competing interests, and more.

Keep your stakeholders central in decision-making. Help others to do the same and to adopt an “outward mindset” focused on others’ needs, objectives, and challenges [11]. Outward mindsets have pervaded ethics and the humanities for millennia—seek wisdom there.

If your stakeholders could see your work and hear the conversations you have about them, how would they feel? Invite them in, talk with them, and enlist their help in co-designing good solutions. Reserve a metaphorical (or real) seat at the table for them in your decision-making.

Assess the diversity of your stakeholders and colleagues. Diversity can help teams be creative, inclusive, and aware of how their work impacts stakeholders. Diversity extends beyond our organizations to finding ways to build win-win capacity and collaborations globally, including across geopolitical and economic barriers.

Help translate data and models to better insights, decisions, and social value. Your work is not done until it has helped others. Be trustworthy and do no harm.

While acting user-first to the best of your ability, be humble about how well you can anticipate all impacts of your work. You can better assess those impacts if you…

## Rule 5: Question your ideas

Critiquing ideas helps improve them. One starting point is building a collaboratively curious culture within your team, especially across disciplinary and organizational boundaries—as is often necessary in complex areas like healthcare and biology. How can you make time to learn from each other [12]?

Heed your wisest critics. Even when you disagree with them, they may see risks and opportunities that you do not. What misuses do your colleagues, regulators, and stakeholders foresee? Can you guard against these misuses? Are there worst-case scenarios to be particularly careful of, such as gain of function research leading to a biosecurity threat?

Consider how you would feel about misuse of your work. It can be eye-opening to play out a “pre-mortem” simulation of a data breach or misuse. Invest in downside prevention and rule and guideline alignment (see Rules 1 and 2).

To help avoid downsides once data science efforts leave your control, design guidance against misuse into the work you deliver, and into live systems via monitoring and alerting. Educate those who will apply your work.

Sometimes the risks you’d like to guard against are best addressed at a sectoral, national, or international level (see Rule 10). Even in these cases, there may be actions that you can take, like growing your colleagues’ risk literacy.

Avoiding downsides is helpful, but you can aim higher. Seek also to…

## Rule 6: Help people live better lives

How we want to influence people shapes the capabilities we build for them. Will your work ultimately help people live, learn, connect, and enjoy?

If you contribute to computational systems that interact with people, you bear some responsibility for how your system influences or constrains people. If you work at a more foundational or basic science level, there is still value in tying your work to a motivating ideal like helping people live better lives. This may steer your work toward higher impact. Get specific where you can—for example, as a biomedical researcher, what are the pathways from your work to better healthspan, cures, or population health?

While it is challenging to understand what leads to a better life, there is a broad overlap in core needs across most societies. Despite our disagreements, we generally agree on many core human needs like reducing child mortality, healing the sick, eating enough, being safe, and having access to basic knowledge [13].

We also differ on important issues [14]. Understand these differences, and how they affect others’ perceptions of your prosocial goals. Where important variation exists, averages can mislead; consider a segmented or personalized approach.

Analyze the benefits from your work and its value tradeoffs. From ratings to simulations to ethics review boards, there are many ways to assess how science and R&D affect core human needs. Can your understanding of what a better life means be deepened by perspectives like those offered by literature and the arts and humanities?

All this may help you build a “humane fitness function” into what you create [15]. Whatever your domain, seek to align your and your organization’s success with genuine human thriving.

To do this, it helps to…

## Rule 7: Work for prosocial leaders and organizations

The more your leaders and organization support genuinely prosocial choices, the more freedom you have to do the same. What commitments have your leaders made? How have they acted prosocially even over short-term benefit to themselves—and made it expected for others to do the same?

Look for prosocial commitments and resourcing from your leaders, while understanding that prosocial cultures differ across academia, government, industry, and civil society. Help your leaders to make prosocial actions part of your organization’s culture. Communicate the costs of not being prosocial, such as the costs of failing at privacy, security, reliability, or public acceptability. Can you co-create a realistic yet inspirational vision for yourself and others [16]?

If you question your leaders’ values, seek first to fully understand them [17]. If fundamental disagreement remains, hard choices await. Will you put aside your personal values, work for change within your organization, or leave? What can you learn from others who faced similar dilemmas?

If you leave, how can you find (or build) an organization in tune with your values? Can you make it easier for others to do the same, such as by sharing assessments of organizations’ level of prosocial behavior?

To align your organization with values in a durable way, you may wish to…

## Rule 8: Build values-aligned business models

The more your organization aligns its success with prosocial goals, the more natural it will be for your leaders and you to pursue such goals. Even if you fail at fully aligning prosocial goals with success, can you improve your organization’s alignment?

Understand the broader societal, business, and legal context that data science lives in. What forces is your organization subject to? If corporate, is there a way to structurally embed prosocial goals into your organization, such as by incorporating under public-benefit legislation or ensuring board and investor alignment? If academic, how can you align the work academics do for their reputation with greater public good and effectiveness [18]? What similar considerations apply in a nonprofit or governmental setting?

To help value alignment, you may wish to seek values-aligned funding. If this proves hard to find, there may be an opportunity to educate funders—or even to help change the worldviews and incentives by which funders operate (see Rule 10). Consider taking part in the ongoing shift to incorporate a broader range of factors into public and private investing and partnerships.

Seek to improve others’ lives without causing harm, while also earning enough value to thrive. That value can come in different forms: reputation and funding in academia, public satisfaction and political support in the public sector, and customer acquisition and retention in the private sector. An example of the latter could be a data-informed diagnostics company that seeks many paying customers as well as broad social benefits (perhaps via sliding payment scales and low unit costs).

Effectively tapping such organizational engines of value—and aligning them with the will to help people live better lives—can drive humane data science applications at scale.

A guideline in doing this is to…

## Rule 9: Leverage algorithmic powers to “raise the floor”

Think of our increasing algorithmic powers as a growing pool of “algorithmic genies” that can serve us—or harm us. Can you leverage these algorithmic genies to “raise the floor” by raising the baseline experience of people worldwide [19]?

Do work that addresses core human needs. Find ways to give a baseline of capabilities to everyone. Then seek to raise that baseline over time.

Raising the floor for core human needs like health (e.g., via reliable health information and diagnostic capabilities for all) is likely to have broad support worldwide. This is increasingly feasible as AI matches more human capabilities in diagnostics and elsewhere, driven by large language models and other advances [20].

We can realistically envision giving everyone access to the equivalent of a personalized diagnostician, educator, and more. Our aim should be not only simply universal access, but also universal guidance and safeguards so that these capabilities lead to better outcomes. This might be supported by measures like mandating guidance on effective use, finding ways to reward AIs and their builders for longer-term outcomes aligned with human well-being, and having a wise human in the loop (such as with genetics and palliative care counselors).

Ensuring that both the least well off and the majority share in the benefits of progress can help raise the floor for all. This kind of “floored progress” seeks ongoing progress for most people, with the constraint that even those who are least well off should reliably reach at least some baseline. Can you seek floored progress from your algorithmic genies and data science, and help their benefits spread widely?

Understand new leverage from emerging superhuman capabilities. Appreciate where these capabilities remain unreliable, and how to align them with human well-being [21]. For example, can you create healthcare solutions that are more rapid, accurate, and accessible? Can you augment rather than replace human capabilities where appropriate, especially where algorithmically augmented humans can thrive while serving others in a richer way than an algorithm alone?

Pursuing the harder goals we have considered may seem daunting. One way to seek positive change at scale is to…

## Rule 10: Collaborate on paradigm shifts

Many aims are far too big to achieve alone. Can you partner with others in bigger prosocial aims—especially ones that “change the rules of the game” to support more win-win outcomes, and make it harder to use data and data science for ill?

For what opportunities are you in the right place at the right time to make a difference? What methods will you draw on, such as codes, coalitions, commitments, laws, and thought leadership? Are your aims best pursued at the level of an organization, industry, region, or worldwide community? What can you learn from those who have grappled with such questions in the business world and elsewhere [22]?

While difficult to do, there can be high leverage in evolving a system’s paradigms: “the mind-set out of which the system—its goals, structure, rules, delays, parameters—arises” [23]. For example, shifting our highest responsibility from short-term profitability to “being a good ancestor” could impact everything from how we invest in global health to the core goals we embed in our systems [24].

Pursuing larger-scale goals can feel like a distraction from your daily work. It helps to find rewards in pursuing larger-scale goals—rewards like enriching your relationships, strengthening your risk and opportunity radar, and boosting your self-respect from building coalitions benefiting all parties and society.

Find the fun in this rule and in the others. Do what you can. Influence others likewise.

These 10 simple rules only begin to articulate what is needed to achieve humane data science. The intent is to help you think about how we might conduct ourselves to achieve the most humane outcomes. Our future depends on it.
